# Moving into the next era of PET myocardial perfusion imaging: introduction of novel ^18^F-labeled tracers

**DOI:** 10.1007/s10554-018-1469-z

**Published:** 2018-10-17

**Authors:** Rudolf A. Werner, Xinyu Chen, Steven P. Rowe, Constantin Lapa, Mehrbod S. Javadi, Takahiro Higuchi

**Affiliations:** 1 Division of Nuclear Medicine and Molecular Imaging, The Russell H. Morgan Department of Radiology and Radiological Science, Johns Hopkins University School of Medicine, Baltimore, MD USA; 2 Department of Nuclear Medicine, University of Wuerzburg, Wuerzburg, Germany; 3 Comprehensive Heart Failure Center, University of Wuerzburg, Oberduerrbacher Strasse 6, 97080 Wuerzburg, Germany; 4 Department of Biomedical Imaging, National Cardiovascular and Cerebral Center, Suita, Japan

**Keywords:** Coronary artery disease, Precision medicine, Positron emission tomography, Myocardial perfusion imaging, ^18^F-flurpiridaz, ^18^F-FBnTP

## Abstract

The heart failure epidemic continues to rise with coronary artery disease as one of its main causes. Novel concepts for risk stratification to guide the referring cardiologist towards revascularization procedures are of significant value. Myocardial perfusion imaging using single-photon emission computed tomography (SPECT) agents has demonstrated high accuracy for the detection of clinically relevant stenoses. With positron emission tomography (PET) becoming more widely available, mainly due to its diagnostic performance in oncology, perfusion imaging with that modality is more practical than in the past and overcomes existing limitations of SPECT MPI. Advantages of PET include more reliable quantification of absolute myocardial blood flow, the routine use of computed tomography for attenuation correction, a higher spatiotemporal resolution and a higher count sensitivity. Current PET radiotracers such as rubidium-82 (half-life, 76 s), oxygen-15 water (2 min) or nitrogen-13 ammonia (10 min) are labeled with radionuclides with very short half-lives, necessitating that stress imaging is performed under pharmacological vasodilator stress instead of exercise testing. However, with the introduction of novel ^18^F-labeled MPI PET radiotracers (half-life, 110 min), the intrinsic advantages of PET can be combined with exercise testing. Additional advantages of those radiotracers include, but are not limited to: potentially improved cost-effectiveness due to the use of pre-existing delivery systems and superior imaging qualities, mainly due to the shortest positron range among available PET MPI probes. In the present review, widely used PET MPI radiotracers will be reviewed and potential novel ^18^F-labeled perfusion radiotracers will be discussed.

**Electronic supplementary material:**

The online version of this article (10.1007/s10554-018-1469-z) contains supplementary material, which is available to authorized users.

## Introduction

The heart failure (HF) epidemic continues to rise with an estimated future financial burden of $70 billion in the year 2030 [1, 2]. Notably, HF has been recently further subclassified into HF with reduced ejection fraction (HFrEF), with preserved ejection fraction (HFpEF), and an intermediate group (HF with mid-range ejection fraction, HFmrEF) [3, 4]. However, one of the main characteristics of either HFrEF, HFpEF or HFmrEF is coronary artery disease (CAD, in up to 54% of the cases) [3, 5, 6] and therefore, its reliable detection, preferably at an early stage of disease, is as relevant as ever [7]. As a result of these considerations, novel strategies for the assessment of flow-limiting coronary artery stenoses have been extensively investigated and myocardial perfusion imaging (MPI) has been an important part of evaluating for this pathology. The most commonly used radiotracers for MPI are the single-photon emission computed tomography (SPECT) agents ^99m^Tc-labeled sestamibi and tetrofosmin, as well as thallium-201 (^201^TI) [8]. In general, the use of positron emission tomography (PET) is expanding worldwide, mainly due to its superior diagnostic performance in oncology [9, 10]. Thus, MPI may benefit from the increasing installed base of latter imaging modality, as PET may provide advantages over SPECT MPI imaging. First, PET has a higher spatiotemporal resolution in comparison to SPECT and a higher count sensitivity. In this light, several studies have already reported on the superior imaging characteristics and higher accuracy of PET MPI compared to conventional SPECT MPI [11, 12]. Moreover, PET includes attenuation correction on a routine basis, as hybrid systems equipped with computed tomography (CT) are routinely installed, which also allows for anatomic co-registration [13]. Apart from that, with traditional PET agents, both rest and stress images can be acquired during one single study, mainly due to the shorter half-life of PET agents [14] and PET has also opened the door for reliable quantification of absolute myocardial blood flow (MBF) [15, 16].

Nonetheless, expensive production procedures with on-site cyclotrons are needed for short-half-life agents [14]. This is in contradistinction to recent developments of novel ^18^F-labeled radiotracers, which may overcome some of the hurdles to adoption of established PET MPI agents. First, ^18^F-labeled imaging probes for MPI may be distributed using delivery systems from central cyclotron facilities. Second, the longer half-life of ^18^F-labeled MPI agents also allows for delayed imaging protocols. From a practical standpoint, exercise stress testing outside of the scanner is feasible [17]. This manuscript reviews this novel class of PET radiotracers for MPI. Among those, ^18^F-flurpiridaz (also previously referred as ^18^F-BMS747158-02) and ^18^F-fluorobenzyltriphenyl-phosphonium (^18^F-FBnTP) have been extensively evaluated and thus, will be further discussed.

## Clinical PET radiotracers for MPI and advantages of ^18^F-labeled radiotracers

To date, the clinically used PET MPI agents are rubidium 82 (^82^Rb, half-life, 76 s), oxygen-15-water (^15^O-water, half-life 2 min) and nitrogen-13-ammonia (^13^N-ammonia, half-life, 10 min) [18]. For the production of ^82^Rb, a commercially available strontium 82 generator is needed, and the high cost for a monthly replacement ($20,000) is a consideration for practitioners as to what extent ^82^Rb PET MPI can be employed in clinical routine [17]. Further drawbacks include its ultrashort half-life and the lowest first-pass extraction (65%) among all available PET MPI agents. In addition, the maximum kinetic energy of positrons emitted during ^82^Rb decay is much higher than that of ^13^N and ^18^F [19]. The latter aspect may have an impact on image quality: high-energy positrons have a long average distance to annihilation and, therefore, the spatial resolution is lower relative to other radionuclides with lower positron energies [17]. The production of ^15^O-water PET depends on a cyclotron unit and it is seen as the gold standard for flow quantification, as it freely diffuses across the cardiomyocyte membrane and produces ideal flow measurements [20]. However, its noisy low-count imaging quality as well as necessity of complex kinetic modeling limits its clinical use [18]. ^13^N-ammonia is approved by the United States Food and Drug Administration and has a very good image quality, but it also requires a costly on-site cyclotron [18, 21].

Notably, use of ^18^F radionuclides may overcome these limitations of commonly used PET MPI radiotracers. Advantages of ^18^F as a radionuclide include, but are not limited to:

(I) ^18^F has a relatively long physical half-life of 110 min, which allows for the use of delivery systems [22] and such an approach has already been proven to be cost-effective for 2-deoxy-2-^18^F-fluoro-d-glucose (^18^F-FDG) [23];
(II) ^18^F has the shortest positron range in tissue compared to other established MPI PET radionuclides [19], and, thus, it may have the highest spatial resolution [17];
(III) the lower positron energy with higher positron yield allows for injection of a considerably lower amount of radioactivity [13];
(IV) its long half-life opens the door for delayed imaging protocols (e.g. for assessment of blood flow alterations at late scan time-points) [24];
(V) due to the short half-life of currently used PET MPI agents, stress imaging is only feasible while placing the patient under pharmacological stress. Notably, ^18^F-labeled radiotracers may overcome this limitation by allowing for physical exercise stress testing outside of the PET device [17, 25, 26].

**Fig. 1:**
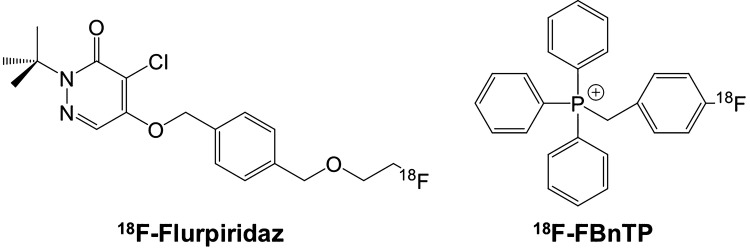
Overview of the herein reviewed ^18^F-labeled PET radiotracers for MPI, namely ^18^F-flurpiridaz and ^18^F-fluorobenzyltriphenyl-phosphonium (^18^F-FBnTP)

To date, the most extensively studied ^18^F-labeled radiotracer for PET MPI is ^18^F-flurpiridaz (Fig. 1).

## ^18^F-labeled radiotracers for MPI: ^18^F-flurpiridaz

### Preclinical evaluation

**Fig. 2:**
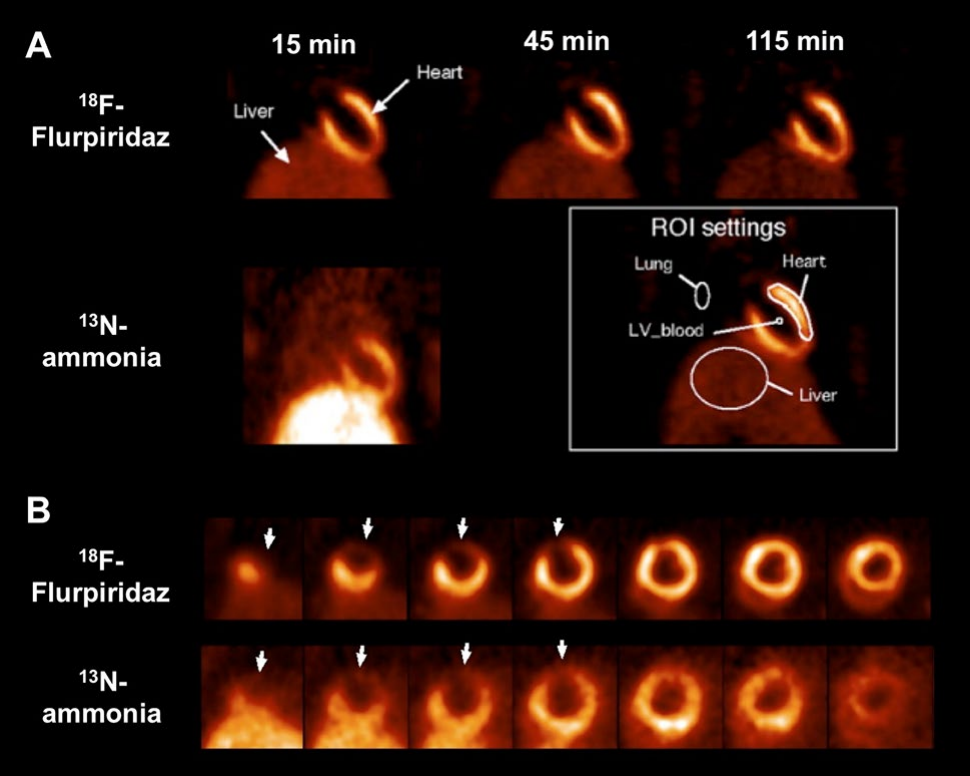
**a** Short-axis ^18^F-flurpiridaz PET in a healthy rat at 15, 45 and 115 min post-injection. The left ventricular myocardium showed excellent contrast to surrounding tissues. ^13^N-ammonia PET at 10 min in a coronal view. Regions of interest placements are displayed in white box. **b** Short-axis images of rat hearts 1 week after coronary artery occlusion using ^18^F-flurpiridaz and ^13^N-ammonia PET. The induced ^18^F-flurpiridaz uptake defect visualized at 15 min corresponded precisely to the defect in ^13^N-ammonia images. However, ^18^F-flurpiridaz demonstrated improved contrast and higher resolution resulting in better delineation of induced lesions, despite a higher injected dose of ^13^N-ammonia (57 MBq) versus ^18^F-flurpiridaz (37 MBq). The inferior/ left ventricular wall can be better distinguished from the liver due to a more rapid liver clearance of ^18^F-flurpiridaz compared to ^13^N-ammonia. Modified from Higuchi et al. [32] © by the Society of Nuclear Medicine and Molecular Imaging, Inc.

**Fig. 3:**
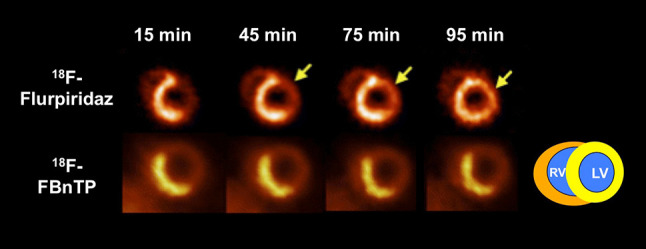
Head-to-head comparison of both ^18^F-labeled myocardial perfusion (MPI) PET radiotracers in a rat model of short-term occlusion and reperfusion. Radiotracers [^18^F-flurpiridaz and ^18^F-fluorobenzyltriphenyl-phosphonium (^18^F-FBnTP)] were injected during ischemia. ^18^F-flurpiridaz showed slow restoration of uptake, while ^18^F-FBnTP remained stable over time, i.e. stability and lack of washout was confirmed for ^18^F-FBnTP [17]. Differences may be explained by different uptake mechanisms of both radiotracers [33]. Modified from Higuchi et al. [32, 33] © by the Society of Nuclear Medicine and Molecular Imaging, Inc

^18^F-flurpiridaz has demonstrated favorable imaging characteristics for MPI in preclinical studies: As a derivative of the pyridazinone insecticide pyridaben, it has a high binding affinity towards mitochondrial complex I, with a considerable high first-pass extraction of > 90% as measured in an isolated perfused heart setup [27, 28]. Comparing ^18^F-flurpiridaz with the established SPECT agent ^99m^Tc sestamibi in a biodistribution rat study, the cardiac uptake of the ^18^F-labeled agent was significantly higher at both early (15 min) and late time points (120 min). This experiment was followed by an isolated rabbit heart perfusion study and net ^18^F-flurpiridaz cardiac uptake increased to a greater extent than that of ^201^TI or ^99m^Tc sestamibi at physiologically relevant flow rates. Moreover, an in vivo PET study demonstrated almost no lung uptake and rapid liver clearance in rats, rabbits, and primates (pronounced washout between 5 and 15 min). In addition, a rat model of coronary occlusion also showed an excellent correlation with ^18^F-flurpiridaz uptake and histopathological findings [29]. These findings were further corroborated in a chronic myocardial infarction (MI) model in rabbits (left coronary artery occlusion, followed by recovery phase over 1 month): compared to controls, a clear defect could be appreciated in the left ventricular wall. The promising safety profile of this imaging agent was further confirmed by electrocardiogram assessments in both controls and MI rabbits [30]. Huisman et al. also used the Langendorff method and investigated the first-pass extraction of ^18^F-flurpiridaz in isolated perfused rat hearts, on which the radiotracer demonstrated a high and flow-independent myocardial first-pass extraction fraction. Thus, ^18^F-flurpiridaz may hold the promise of a linear correlation between radiotracer uptake and cardiac blood flow [28]. Higuchi and coworkers tested ^18^F-flurpiridaz in rats in vivo. Normal healthy control rats were found to have a homogoneous delineation of the myocardium up to 2 h after tracer injection. However, for the permanent occlusion model, the defect size remained stable over the entire imaging protocol (15–115 min). This was in contradistinction to the transient ischemia model: reperfusion after short, transient ischemia of 3 min showed radiotracer redistribution to the induced defect (i.e. tracer redistribution after reperfusion). Radiotracer reinjection further enhanced the normalization process. The concept of redistribution is based on underperfused but viable myocardium, which retains the radiotracer while it washes out of normal myocardial areas, i.e. initial defects appear to normalize [31]. The clinical application are diagnosis of CAD and most importantly, for the assessment of tissue viability, e.g. by radiotracer injection under physical stress with early and delayed imaging protocols, which allows to monitor such redistribution closely over time. Figure 2 shows the superior imaging characteristics of ^18^F-flurpiridaz PET compared to a common PET MPI agent, ^13^N-ammonia, in (A) healthy rats and (B) in a rat model after coronary artery occlusion. The ^18^F-labeled radiotracer demonstrated improved contrast and higher resolution, resulting in better delineation of induced lesions, despite a higher injected dose of ^13^N-ammonia relative to ^18^F-flurpiridaz. For the ^18^F-labeled imaging agent, the inferior/left ventricular wall can be better distinguished from the liver [32]. Figure 3 displays a head-to-head comparison of ^18^F-flurpiridaz and ^18^F-FBnTP in a rat model of short-term occlusion and reperfusion. For the latter radiotracer, retention stability over time was confirmed, while ^18^F-flurpiridaz showed slow restoration over time. Differences may be explained by the underlying uptake mechanisms: ^18^F-flurpiridaz targets mitochondrial complex I, while ^18^F-FBnTP localizes to mitochondria due to membrane potential [33]. The observed kinetics (redistribution after reperfusion) may allow for the use of ^18^F-flurpiridaz in a similar way to clinical protocols for the diagnosis of CAD with conventional stress/rest ^201^TI perfusion protocols or for the assessment of myocardial viability [32, 34]. In a permanent and transient occlusion model of the left coronary artery, uptake defect assessed by ^18^F-flurpiridaz closely correlated with histological measured scar sizes confirmed by 2,3,5-triphenyltetrazolium chloride staining [35]. In a pig model, Guehl et al. demonstrated that accurate rest and stress blood flow estimations with ^18^F-flurpiridaz are feasible, even in less than 15 min of PET acquisition time by using a single-scan rest-stress method, which further emphasizes the practicality of this radiotracer in clinical routine [36]. Also in a pig model, Sherif et al. showed that ^18^F-flurpiridaz retention and standardized uptake values (SUVs) correlated with absolute MBF values at rest and pharmacological stress. As such, SUVs may be used as a substitute for absolute blood flow. As SUV does not require determination of radiotracer input function, tracer injection and exercise treadmill or bicycle stress test protocols could be performed outside the scanner. From a practical standpoint, such an approach may facilitate flow estimation in clinical routine [37]. By comparison with radioactive microsphere-derived blood flow in a pig model, a high agreement rate with regional MBF using ^18^F-flurpiridaz was achieved, even over a wide flow range [38].

### Clinical studies

**Fig. 4:**
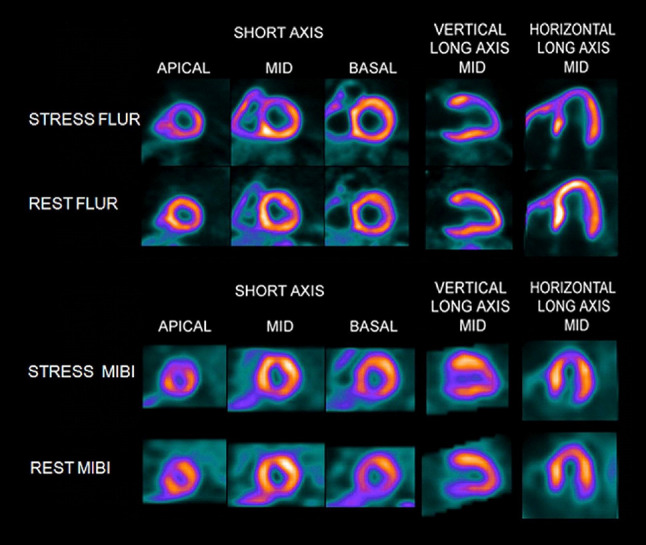
FLUR PET and MIBI SPECT images from an 82-year-old man. The FLUR PET (top) and MIBI SPECT (bottom) images from an 82-year-old man with shortness of breath and an occluded native proximal left anterior descending (LAD) coronary artery and an occluded left internal mammary graft to the LAD and no other significant native CAD. The FLUR images show a severe reversible perfusion defect throughout the territory of the occluded proximal LAD, whereas the MIBI images show only a moderate perfusion defect in the distal LAD territory (apical slices). FLUR = Flurpiridaz F 18; MIBI = Tc-99m sestamibi. Reprinted from the Journal of the American College of Cardiology (JACC), 61(4), Daniel S. Berman, Jamshid Maddahi, B. K. Tamarappoo, Johannes Czernin, Raymond Taillefer, James E. Udelson, C. Michael Gibson, Marybeth Devine, Joel Lazewatsky, Gajanan Bhat, Dana Washburn, Phase II safety and clinical comparison with single-photon emission computed tomography myocardial perfusion imaging for detection of coronary artery disease: flurpiridaz F 18 positron emission tomography, 469-77, Copyright (2013), with permission from Elsevier [41]

In a phase I trial enrolling healthy volunteers, a sustained retention was recorded up to 5 h post-injection and the radiotracer was well tolerated in all 13 subjects [39]. Clear and homogenous delineation of the myocadium was appreciated up to 5 h after administration, while liver clearance was observed 2 h post-injection [39]. Thus, the radiopharmaceutical is present in the myocardium to allow for an administration of the radiotracer at peak treadmill exercise. Moreover, kinetic studies demonstrated that imaging can be performed immediately after completing the exercise protocol and thus, ^18^F-flurpiridaz may identify even subtle stress-induced wall motion abnormalities (compared to SPECT with ^99m^Tc agents, which generally involve imaging at least 30 min post-injection) [25]. Apart from that, Packard et al. enrolled seven healthy subjects with a low likelihood of myocardial ischemia and 8 CAD patients using ^18^F-flurpiridaz. Notably, such a study design provided a wide range of MBF. In patients with no stress-inducible ischemia, no significant differences in MBF (either at rest or adenosine stress) and myocardial flow reserve (MFR) were recorded. This was in contradistinction to CAD subjects: lower MBF in diseased vascular segements after adenosine stress was noted and therefore, also a reduction in MFR [40]. Berman et al. evaluated the efficacy and safety profile of ^18^F-flurpiridaz in a phase II trial. In 143 subjects from 21 different study sites, stress-rest PET and ^99m^Tc sestamibi SPECT were performed, while the latter imaging modality served as a comparator. The certainty of interpretation, which was recorded by three blinded readers in a binary fashion (abnormal vs. normal), was considerably higher for PET (90.8% vs. SPECT, 70.9%). In 86 patients, who also underwent invasive coronary angiography (ICA, as a reference standard for coronary stenosis), PET revealed a higher sensitivity compared to SPECT, while specificity remained similar. Of note, in patients suffering from CAD (detected on invasive procedures), the magnitude of the reversible defect assessed with PET was larger than with SPECT. Figure 4 displays a mismatch of ^18^F-flurpiridaz PET MPI versus ^99m^Tc-sestamibi SPECT MPI in an 82-year old male with an occluded native proximal left anterior descending (LAD) coronary artery and an occluded left internal mammary graft to the LAD. On ^18^F-flurpiridaz PET MPI, a reversible perfusion defect throughout the territory of the occluded proximal LAD was noted; the ^99m^Tc-sestamibi images showed only a moderate perfusion defect in the distal LAD territory [41]. Assessing the summed difference score for ^18^F-flurpiridaz MPI and ^99m^Tc-sestamibi MPI, stress induced perfusion abnormalities in patients with multivessel CAD were significantly higher with PET MPI [42]. Recently, Bateman et al. reported on 795 subjects from 72 international sites and described previous results of the first Phase III trial. The authors noted superior diagnostic performance characteristics for ^18^F-flurpiridaz relative to SPECT MPI for the assessment of CAD in obese patients [43]. The recently launched, prospective, international, multi-center, open-label AURORA study (second Phase III study, ClinicalTrials.gov Identifier: NCT03354273) will include subjects with suspected CAD scheduled for ICA and both SPECT and PET MPI will be carried out prior to intervention. The primary endpoint is diagnostic efficacy of ^18^F-flurpiridaz PET MPI in detecting significant CAD [44, 45].

## ^18^F-labeled radiotracers for MPI: ^18^F-FBnTP

The lipophilic cation ^18^F-FBnTP also accumulates in myocardial mitochondria [46]. In mongrel dogs, uptake and retention kinetics were tested in vivo and ^18^F-FBnTP reached its plateau in the left ventricle 5 min after radiotracer administration. A delineation of the myocardium was still seen 90 min post-injection. In addition to that, the metabolite concentration in the blood was considerably low and the heart-to-liver ratio was 1.2 after 60 min. The heart-to-lung ratio was 12:1 (5 min post-injection), which was much higher than reported for ^99m^Tc-labeled SPECT agents (2:1) in the same species. Thus, one may speculate that the lower background activity leads to better imaging contrast relative to SPECT counterparts [47, 48]. In addition, ^18^F-FBnTP was also compared to ^99m^Tc-tetrofosmin SPECT in vivo by using various degrees of coronary artery stenosis: 17 dogs with different degrees of stenosis of LAD or circumflex coronary arteries were enrolled. Microsphere flow was assessed with radioactive micropsheres, which allow for distinction of true myocardial blood flow in ischemic versus non-ischemic beds of the left ventricle. Compared to ^99m^Tc-tetrofosmin, superior diagnostic performance for ^18^F-FBnTP was reported, in particular for the assessment of mild or severe stenosis [49]. To reveal further insights into kinetics of ^18^F-FBnTP, short transient coronary artery occlusion (ligation of the left coronary artery, 2 min) was induced in Wistar rats, which was followed by reperfusion. PET imaging with ^18^F-FBnTP showed that the radiotracer remained stable demonstrating no washout or redistribution and matched histologically proven defect areas [33]. Recently, in a rat model of autoimmune myocarditis, the longitudinal imaging characteristics of ^18^F-FDG were investigated and ^18^F-FBnTP was used as a reference perfusion marker [50]. Albeit this radiotracer is used in a preclinical setting over the last years, human data are still lacking and thus, if a more widespread adoption is envisaged, further clinical trials are warranted. In addition to ^18^F-FBnTP, 18F-labeled fluoroalkylphosphonium derivatives (^18^F-FATPs) have been synthesized as well: these are (5-^18^F-fluoropentyl)triphenylphosphonium cation (^18^F-FPTP), (6-^18^F-fluorohexyl)triphenylphosphonium cation (^18^F-FHTP), and (2-(2-^18^F-fluoroethoxy)ethyl)triphenylphosphonium cation (^18^F-FETP). Compared with ^13^N-ammonia in a rat model of coronary occlusion, ^18^F-FATPs showed excellent image quality, along with rapid liver and lung clearance [51].

Table 1 summarizes key properties of ^18^F-labeled radiotracers for PET MPI. Supplementary Table 1 displays characteristics of established PET MPI agents and the novel PET agent ^18^F-flurpiridaz.

**Table 1:** Advantages and limitations of the reviewed ^18^F-labeled PET radiotracers for MPI, namely ^18^F-flurpiridaz and ^18^F-fluorobenzyltriphenyl-phosphonium (^18^F-FBnTP)

^18^F MPI PET radiotracers	Advantages	Limitations
^18^F-flurpiridaz	• Considerable high first-pass extraction of > 90% [27, 28] • Almost linear correlation between tracer uptake and cardiac blood flow in isolated perfused rat hearts [28] • Radiotracer redistribution after reperfusion, i.e. ^18^F-flurpiridaz may be suitable for clinical protocols similar to conventional stress/rest ^201^TI perfusion protocols or assessment of myocardial viability [32] • Phase I: radiotracer present up to 5 h post-injection, i.e. injection at peak treadmill exercise is feasible [39] • Phase II: compared to stress-rest SPECT MPI, PET MPI with superior performance characteristics for overall CAD diagnosis [41] • First Phase III study: superior perfusion defect detection of ^18^F-flurpiridaz relative to SPECT MPI for the assessment of CAD in obese subjects [43] • Second Phase III study (AURORA): will assess the efficacy of ^18^F-flurpiridaz PET MPI in detecting significant CAD compared to SPECT MPI in patients scheduled for invasive coronary angiography [44, 45]	• Limited to academic centers • Cyclotron production • No FDA approval yet • Cost-effectiveness data are lacking
^18^F-FBnTP	• Superior diagnostic performance for ^18^F-FBnTP compared to SPECT MPI in dogs [49] • Lack of redistribution in a rat model of short transient coronary artery occlusion (2 min) and reperfusion (i.e. tracer injection remote from the imaging device may be feasible, e.g. in a chest pain unit) [33]	• No larger clinical trials • Limited to academic centers • Cyclotron production • Cost-effectiveness data are lacking

*SPECT* single photon emission tomography, *CAD* coronary artery disease, *FDA* Food and Drug Administration

## Future directions

^18^F-labeled radiotracers allow for an improved target-to-background ratio compared to commonly used PET MPI agents, which in turn leads to higher imaging quality [32]. Thus, given the superior imaging characteristics of ^18^F-labeled PET MPI radiotracers compared to other SPECT or PET MPI competitors, it is possible that these novel radiotracers further contribute to an even more tailored treatment approach for ischemic heart patients. Notably, the extraction fraction of those radiotracers at various flow rates open the door for optimal absolute MBF quantification [40]. Cutoff values of both MBF and MFR could be established with those radiotracers and thus, could be used for risk stratification [40]. This would apply to different subgroups in a clinical context which are at higher risk of cardiac events, such as diabetes or chronic kidney diseases [52]. The latter group is of great interest, as cardiovascular disease is the main cause of death among patients suffering from severe renal dysfunction [53]. However, conventional SPECT MPI cannot identify high-risk patients across a wide spectrum of renal (mal)function and thus, novel approaches using ^18^F PET MPI radiotracers may have an increased prognostic capability [54]. In addition, quantification of MBF (assessed by ^82^Rb PET) in patients prior to heart transplantation can also identify subjects at high risk of suffering from later clinical events [55]. However, the longer half-life of ^18^F PET MPI along with their superior imaging quality may allow for a more practical adoption in clinical routine and a more thorough evaluation of the perfusion status in heart transplant recipients. Other applications of such radiotracers include chest pain with normal findings on coronary angiography [52]. In a similar vein like for MPI PET agents, a recent shift from established cardiac neuronal PET agents (^11^C-hydroxyephedrine) towards novel ^18^F-labeled PET tracers to measure cardiac nerve integrity has been noted, e.g. by the use of the myocardial nerve imaging agent ^18^F-LMI1195 [56]. Thus, in a dual-tracer approach, both newly introduced ^18^F radiotracers (^18^F-flurpiridaz for MBF and ^18^F-LMI1195 for cardiac nerve integrity) could be used. Such a global functional assessment of the heart has been also previously tested in a rat model of ischemia (with ^11^C-HED and ^201^TI for perfusion): compared to the perfusion defect areas, a larger ^11^C-HED uptake defect in both subacute and chronic phases was noted [57]. Thus, further clinical applications, preferably with ^18^F-labeled cardiac perfusion/nerve tracers, which offer superior imaging quality, would be of great interest.

## Conclusions

^18^F-labeled radionuclides for PET MPI perform well in assessing the defect size in CAD patients. First, they are less expensive to produce and may also be distributed using delivery systems from central cyclotron facilities [23]. Second, the longer half-life of ^18^F-labeled MPI agents also allow for delayed imaging protocols, which in turn may allow for physical exercise stress testing protocols outside of the scanner [17]. In light of its excellent extraction fraction, ^18^F-flurpiridaz has very favorable characteristics as a PET MPI agent and phase II/III trials have reported on a superior diagnostic performance relative to common SPECT MPI agents [39, 41, 43]. In the currently recruiting AURORA trial, subjects referred for ICA because of suspected CAD will undergo both SPECT and PET MPI prior to intervention [44, 45]. The results may reveal further insights into the efficacy of ^18^F-flurpiridaz PET MPI in detecting significant CAD. However, MPI (either with PET or SPECT) still remains underrepresented in some countries: for instance, in Germany, CAD diagnosis seems to be mainly shifted directly to invasive angiographic procedures, which in turn leads to less requests of such tests in clinical routine [58].

## Electronic supplementary material

Below is the link to the electronic supplementary material.

Supplementary material 1 (DOCX 22 KB)
