# Supplementary material for: Moving into the next era of PET myocardial perfusion imaging: introduction of novel 18F-labeled tracers
Source: Int J Cardiovasc Imaging. 2018 Oct 17;35(3):569–77. doi: 10.1007/s10554-018-1469-z (PMC6454078; doi:10.1007/s10554-018-1469-z)
Supplement: Supplementary file 1 — Supplementary material 1 (DOCX 22 KB) [file 10554_2018_1469_MOESM1_ESM.docx]

**Moving into the Next Era of PET Myocardial Perfusion Imaging - Introduction of Novel 18F-labeled Tracers**

Rudolf A. Werner^1,2,3^, Xinyu Chen^2,3^, Steven P. Rowe^1^, Constantin Lapa^2^, Mehrbod S. Javadi^1^, Takahiro Higuchi^2,3,4,#^

1. Division of Nuclear Medicine and Molecular Imaging, The Russell H. Morgan Department of Radiology and Radiological Science, Johns Hopkins University School of Medicine, Baltimore, MD, United States;
2. Department of Nuclear Medicine, University of Wuerzburg, Wuerzburg, Germany;
3. Comprehensive Heart Failure Center, University of Wuerzburg, Wuerzburg, Germany;
4. Department of Biomedical Imaging, National Cardiovascular and Cerebral Center, Suita, Japan.

^#^ Corresponding Author

*Running title:* ^18^F PET MPI

*Article Type:* Review

*Address for correspondence:*

Takahiro Higuchi, M.D. PhD.

Department of Nuclear Medicine/Comprehensive Heart Failure Center,

University of Wuerzburg, Oberduerrbacher Strasse 6, 97080 Wuerzburg, Germany.

Mail: [thiguchi@me.com](mailto:thiguchi@me.com), fax: +49 931 201 6 444 00, phone: +49 931 201 35455.

|  | ^82^Rb | ^15^O-water | ^13^N-ammonia | ^18^F-flurpiridaz |
| --- | --- | --- | --- | --- |
| Half-life | 76 sec | 2 min | 10 min | 110 min |
| Positron Range in tissue in mm | 8.6 | 4.14 | 2.53 | 1.03 |
| Maximum energy in MeV* | 3.38 | 1.74 | 1.2 | 0.64 |
| Imaging Quality | lowest | intermediate | intermediate-high | highest |
| Production | Generator | On-site cyclotron | On-site or nearby cyclotron | Central cyclotron  facility |
| Myocardial extraction fraction in % | 65 | 100 | >80 | >90 |
| Preferred Stress Testing | Pharmacological  Stress | Pharmacological  Stress | Pharmacological Stress; Physical Exercise Stress is feasible, but challenging | Physical  Exercise Stress |
| Estimated Radiation Dose in mSv | 2.52 | 2.05 | 3.07 | 6.4 |

**Supplementary Table 1. Key characteristics of the established positron emission tomography (PET) radiotracers for myocardial perfusion imaging (MPI) and the novel PET MPI probe ^18^F-flurpiridaz.** ^82^Rb = Rubidium 82, ^15^O-water = Oxygen-15-Water, ^13^N-ammonia = Nitrogen-13-Ammonia, ^18^F-flurpiridaz = also commonly referred as ^18^F-BMS747158-02. MeV = megaelectronvolts [19,26]. * High-energy positrons have a considerable long travel distance prior to annihilation and therefore, the spatial resolution is lower relative to other, low-energy radionuclides [17].
